# Case Report: Pembrolizumab in a patient with preexisting paraneoplastic dermatomyositis and sarcomatoid urothelial carcinoma. Searching for balance

**DOI:** 10.3389/fimmu.2025.1558964

**Published:** 2025-03-17

**Authors:** Mariangela Torniai, Giuseppe Pio Martino, Calogero Gucciardino, Stefano Angelici, Renato Bisonni

**Affiliations:** ^1^ Unità Operativa Complessa (UOC) Oncologia, Ospedale Murri, Fermo, Italy; ^2^ Unità Operativa Complessa (UOC) Medicina Interna, Ospedale Murri, Fermo, Italy

**Keywords:** immunotherapy, paraneoplastic syndrome, autoimmune disease, dermatomyositis, urothelial carcinoma, pembrolizumab

## Abstract

Dermatomyositis (DM) is an uncommon systemic autoimmune disorder classified as one of the idiopathic inflammatory myopathies (IIM). DM could also represent a manifestation of an underlying neoplasm with a relative risk of cancer globally ranging from 3% to 8%. Owing to the strong connection between immunosurveillance and cancer progression, the management of paraneoplastic DM represents a challenging issue. To complicate matters is the advent of cancer immunotherapy, that might interfere with self-tolerance with a true risk of previous autoimmune disorders re-exacerbation. We report the case of a 50-year-old patient with advanced urothelial bladder cancer and preexisting paraneoplastic DM treated with pembrolizumab. On the basis of our experience, previous paraneoplastic DM might not necessarily represent an absolute contraindication for ICIs treatment. Furthermore, this case might suggest a role of intravenous immunoglobulins (IVIG) in preventing DM reactivation, underling the importance of a multidisciplinary approach.

## Introduction

Immune checkpoint inhibitors (ICIs) represent a relatively new class of antineoplastic drugs approved to treat many cancer types, including urothelial carcinoma, with durable long-term responses and a favorable toxicity profile (1–3). ICIs have relocated the paradigm of cancer treatment acting through the stimulation of immune system to fight tumor cells. These monoclonal antibodies work by blocking interaction between a group of transmembrane molecules (as programmed cell death protein, PD-1) and their ligands (as PD-L1 and PD-L2), normally responsible of T-cells exhaustion, immune response modulation and self-tolerance in order to maintain immunologic homeostasis (4). Due to their mechanism of action, ICIs show a very specific safety profile with a considerable variety of immune-related adverse events (irAEs) triggered by T-cells activation. irAEs management require a prompt identification and grading, an adequate diagnostic work-up and, most important, a specific treatment according to the severity of toxicity (5). On this basis, underlying autoimmune disorders (including autoimmune paraneoplastic syndrome) have constantly represented a factor precluding inclusion in cancer immunotherapy clinical trials. Therefore, real-world evidence is essential to acquire data regarding ICIs efficacy and safety in this specific population (6). Here we present the case of preexisting paraneoplastic DM in a patient with urothelial bladder cancer who received ICI (pembrolizumab) for advanced disease, with a review of other reported cases.

## Case description

**Figure 1:**
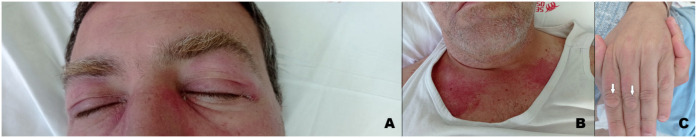
Clinical manifestation of dermatomyositis: **(A)**. Violaceous rash over the eyelids with periorbital oedema (heliotrope rash). **(B)**. Erythematous macules involving the anterior aspect of the neck and the upper chest (V sign). **(C)**. Papules overlying Interphalangeal joints of the hand (Gottron’s papules).

A 50-year-old man with no significant comorbidities and a smoking history of over 50 pack-years presented with 3 months progressive fatigue, muscles weakness and diffuse joint pain (especially in both hips and shoulders) associated with heliotrope rash (**Figure 1A**), photosensitivity with purplish sunburn reaction over décolleté and anterior chest (**Figure 1B**) and papules overlying Interphalangeal joints of the hands (Gottron’s papules) (**Figure 1C**). Laboratory evaluation showed normocytic anemia, transaminitis and a creatine phosphokinase (CPK) level of 3500 units/L (normal 26–192 UI/L). Electromyography and muscle biopsy supported the preliminary diagnosis of inflammatory myopathy. Screening for specific antibodies of myositis were positive for anti-transcription intermediary factor 1 gamma (anti-TIF1-γ) corroborating the diagnosis of DM. The patient underwent whole-body computed tomography (CT) with IV contrast that was negative for neoplastic diseases. He received IV high-dose corticosteroids with no response. A 3-month second-line therapy course of IVIG at a dose of 2.0 g per kilogram of body weight were administrated leading to a prompt reduction of both CPK level and DM clinical manifestations. Prednisone was tapered over 8 weeks. About six months later the diagnosis of DM, the patient suddenly presented gross hematuria with severe anemia, hypotension and suprapubic pain, all coinciding with a mild exacerbation of cutaneous and muscular manifestation of DM as heliotrope rash and muscles weakness and pain requiring an increase of daily prednisone. An abdominal CT with IV contrast showed irregular bladder wall thickening with foci of urothelial hyperenhancement and a vascularized mass along posterior wall, several enlarged pelvic lymph nodes, two suspected liver metastases in the right lobe and also a bone metastasis in left ischiopubic ramus. Bladder biopsy taken during cystoscopy demonstrated urothelial carcinoma. Cancer staging was completed with brain and thoracic CT, showing negative results, and 18F-fluorodeoxyglucose positron emission tomography (FDG-PET) that confirmed locoregional nodal, bone and liver metastasis. Due to intractable hematuria, the patient underwent palliative radical cystectomy, lymphadenectomy and Bricker-type cutaneous ureteral ileostomy, with diagnosis of sarcomatoid urothelial carcinoma infiltrating the entire bladder wall and local lymph nodes (stage pT4N2). One month after surgery, he was started on first-line chemotherapy with cisplatin and gemcitabine with concomitant administration of pegfilgrastim and dexamethasone during each cycle. We knew that our patient would be candidate to receive avelumab as maintenance therapy or pembrolizumab as second-line treatment subsequently chemotherapy; therefore, after multidisciplinary discussion involving rheumatologist and medical oncologist as well the patient itself, we decided to administrate another 3-month course IVIG preceding ICI initiation to reduce the risk of DM exacerbation as well as steroid sparing agent to avoid any potential loss of efficacy. In the course of chemotherapy DM symptoms and laboratory tests (CPK and inflammatory markers) gradually improved also due to IVIG administration and daily prednisone was gradually tapered to < 10 mg. A whole-body CT scan performed after four cycles revealed metastatic progression in bone (right ischiopubic ramus and iliac wing) and lungs with round sharply nodules of varying size throughout the pulmonary fields. Due to disease progression, the patient started pembrolizumab continuing with the administration of IVIG to prevent DM exacerbation. During immunotherapy the patient was closely monitored to assess the onset of irAEs: physical examination, kidney, liver and pancreatic function tests together with thyroid and pituitary gland function tests and CPK were performed before each ICI administration, while echocardiogram was done every 3 cycles. Pembrolizumab was well tolerated: the patient was totally asymptomatic and did not report any irAEs as well as any exacerbation of myopathy symptoms and skin manifestation related to DM. The first CT scan performed after the fourth cycle showed stable disease; on the assumption of good tolerance and efficacy, the patient continued pembrolizumab and concomitant IVIG administration. Unfortunately, six months after starting immunotherapy the patient re-experienced heliotrope rash around eyes and on both cheeks and erythema over chest and upper back, followed by muscle weakness of the extremities and myalgia without oculomotor involvement; serum CPK restarted growing. DM exacerbation required higher dose of steroids; at the same time, pembrolizumab was discontinued and a whole-body CT scan was performed showing cancer progression in liver. Therefore, the patient started treatment with enfortumab vedotin (EV) continuing with concomitant administration of high-dose corticosteroids and IVIG due to DM exacerbation. Two days after EV first administration, he experienced a severe worsening of skin manifestation involving all the extremities, extreme muscle weakness, difficulties in walking and swallowing. Supportive therapy with fluids, parenteral nutrition and intravenous higher dose of steroids were administrated achieving a gradually improvement of clinical condition within four weeks. A whole-body CT scan showed further disease progression with new bone lesions in the T12-L2 vertebral bodies, lymph node metastasis in the mediastinal and abdominal fields and major metastatic dissemination in lungs. The patient received fourth line therapy with weekly paclitaxel but he passed away two months after starting chemotherapy.

## Discussion

**Figure 2:**
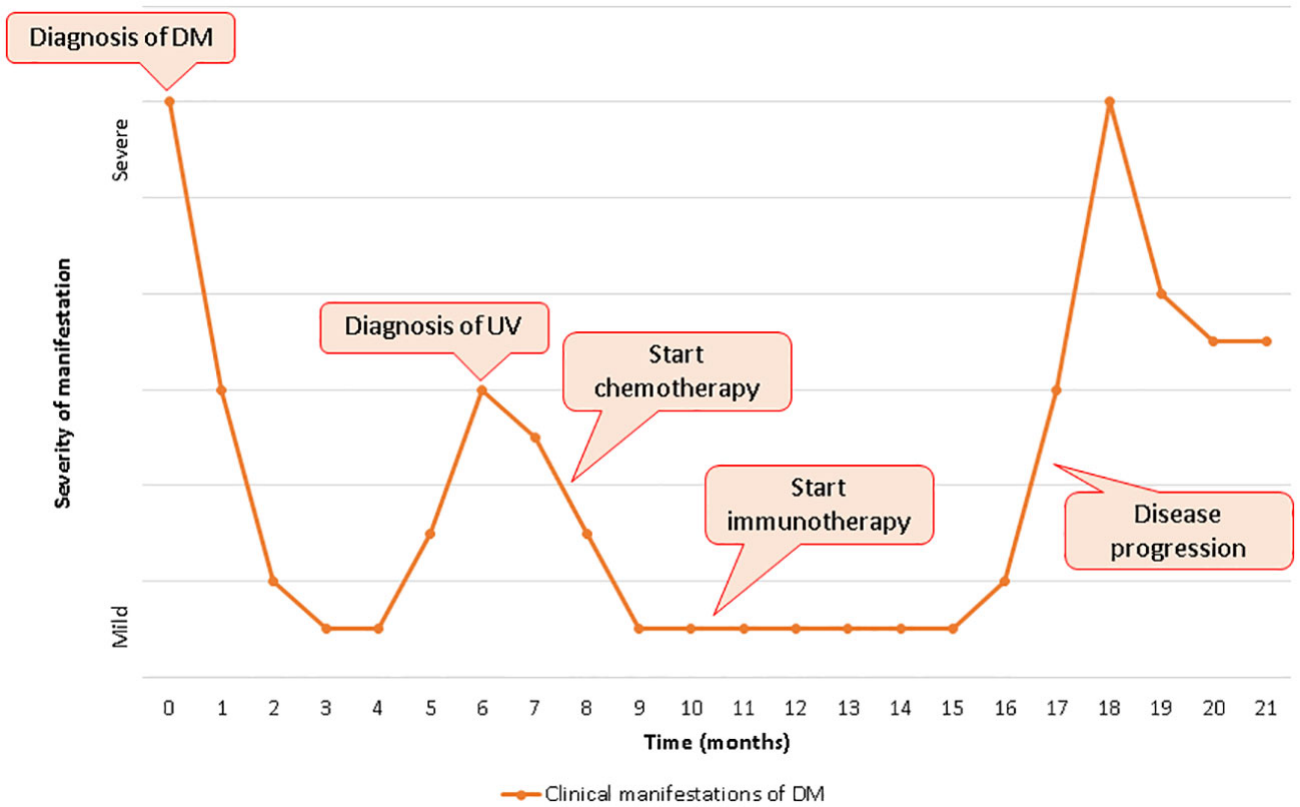
Clinical manifestation of dermatomyositis and clinical course of bladder carcinoma over time.

DM is an uncommon systemic autoimmune disorder of unknown pathogenesis and classified as one of the IIM. In adults, the disorder is characterized by chronic inflammation of the skin and muscles leading to rashes and progressive weakness, predominantly in proximal muscles. In 2017, the European League Against Rheumatism and the American College of Rheumatology (EULAR/ACR) released a set of criteria to help identify IIM and its major subgroups. According to this classification, a combination of clinical criteria, laboratory tests and muscle biopsy are used to calculate the probability of having IIM (7). The presence of characteristic skin findings in addition to symmetric muscle weakness should help to distinguish DM from other conditions (inclusion body myositis, drug-induced myopathy, hypothyroidism, polymyalgia rheumatica, muscular dystrophies, motor neuron disease, neuropathy, inherited metabolic myopathies and myasthenia gravis). Electromyography might help to differentiate DM from neuropathic causes of weakness. Muscle biopsy showing the hallmark pathological features of DM also helps to exclude other causes. Glucocorticoids are administered as first-line therapy, followed by various immunosuppressants; IVIG usually in combination with immunosuppressive drugs and has been recommended, also as a glucocorticoid-sparing agent in patients with this disorder. More recently, in a pivotal phase III, double blind, parallel group, randomized placebo-controlled trial, IVIG achieved a significant clinical response based on a composite score of disease activity compared to placebo (79% versus 44%) at 16 weeks (8). The advantages of IVIG include safety in pregnancy, malignancy, and absence of infectious risk (9). DM could also represent a manifestation of an underlying neoplasm, appearing as a facultative paraneoplastic syndrome with a relative risk of cancer globally ranging from 3% to 8%. This association seems stronger in male and older (> 45 years) patients, while juvenile DM rarely constitutes a paraneoplastic condition. DM diagnosis might precede (with a higher risk in the first year after the onset), follow or be concomitant to tumor detection; therefore, a comprehensive cancer screening should be accomplished in all patients, although no specific guidelines are available to date (10). Since TIF1-γ antibody have been discovered in 83% of patients with paraneoplastic DM, Ferronato M et al. recently suggested the importance of TIF1- γ, a transcript regulator involved in DNA damage repair and tumor suppression, in the pathogenesis of cancer-associated DM acting as a tumor autoantigen. However, a genetic predisposition should not be ignored considering the strong association between this syndrome and specific HLA sequences (HLA-DRB1*0301 and HLA-DQA1*0501 in Caucasians), regardless of dysregulated expression of TIF1- γ (11). Many types of malignancy might be associated to this autoimmune disease, especially gastrointestinal, ovarian and lung carcinomas, with a significant heterogeneity reflecting cancer prevalence across different population (12). In 1982 Behan WMH et al. firstly reported a case of paraneoplastic DM in a 69 years old patient with localized bladder carcinoma and other concomitant autoimmune disorders (myasthenia gravis, Hashimoto’s thyroiditis and pemphigoid), suggesting the importance of both disordered immunoregulation and genetic predisposition in the pathogenetic mechanisms (13). From then to 2023, at least 38 other cases of paraneoplastic DM in UC were published worldwide, with a median age of the patients equal to 67.5 years and a mighty predominance in males than females. In many cases UC was localized and treated with only surgical resection, and the most common histological type was transitional cell carcinoma; among patients tested for antibodies, only 20% was positive for antinuclear antibody (ANA) was positive (14, 15). Treatment of paraneoplastic DM is similar to non-cancer-associated forms although few peculiarity: (1) long-term immunosoppressive therapy might promote cancer progression and/or cancer relapse weakening immunosurvelliance; (2) interactions between antineoplastic drugs and DM treatment are common at both pharmacokinetic (interference in hepatic metabolic pathways with increased toxicity) and pharmacodynamic (concomitant adverse effects) level; (3) antitumor agents might cause adverse events simulating DM symptoms (i.e. weakness for paclitaxel, skin rash for capecitabine/5-fluorouracil, *etc.*); (4) tumor response frequently leads to a concomitant improvement of paraneoplastic DM (16). Therefore, the management of cancer-related DM represents an arduous challenge requiring a multidisciplinary approach and a close collaboration between physicians of different specialties (*in primis* oncologist and rheumatologist). Furthermore, the advent of cancer immunotherapy in the treatment of a huge types of neoplasms raised the stakes. Due to their mechanism of action, ICIs might interfere with self-tolerance inducing autoimmunity as an unpredictable side effect. Skin toxicity (including autoimmune disorders) represents the most expected reaction occurring in > 50% of treated patients although usually not severe (17). Other common irAEs includes endocrinopathies, hepatic and gastrointestinal toxicity and lung toxicity, while immuno-related myositis is a rare (< 1%) but potentially fatal complication of ICIs requiring rapid treatment with high-dose corticosteroids possibly followed by immunosuppression, plasmapheresis or IVIG in severe cases (18). Even though their uncommon occurrence, immuno-related DM have been already documented in different type of neoplasms (melanoma, small cell lung cancer, lung adenocarcinoma, renal-cell carcinoma, non-Hodgkin lymphoma) treated with different type of ICIs (ipilimumab, nivolumab, pembrolizumab, cabiralizumab + nivolumab). In all cases immunotherapy was discontinued and all patients required corticosteroids with or without other IVIG and immunosoppressive agents (19). DM in oncologic patients receiving ICIs might represent a paraneoplastic syndrome as well as a treatment-related adverse event. An early onset (antecedent to immunotherapy) and the presence of anti-TIF1-γ antibodies generally suggest the diagnosis of cancer-related DM, although a preexisting paraneoplastic syndrome might be worsened by antineoplastic drugs (20). Due to their safety profile, patients with underlying autoimmune disorders (including autoimmune paraneoplastic syndrome) were excluded from cancer immunotherapy pivotal trials. Therefore, data regarding ICIs efficacy and safety in this specific population remain uncertain and mainly resulting from real-world setting. In their retrospective study, Kehl KL et al. showed that immunotherapy can reactivate preexisting autoimmune disease (in about 40% of cases) but also promote the development of new autoimmune manifestations with a modest increase of hospitalization and corticosteroid treatment (21). Furthermore, a higher risk of toxicity with a shorter irAEs-free survival time was observed in another retrospective case series (6). Although prudence is recommended, previous autoimmune disorders might not necessarily represent an absolute contraindication for ICIs treatment whenever patients are strictly monitored (6, 21). In our patient, paraneoplastic DM preceded the diagnosis of urothelial carcinoma figuring a cancer-related syndrome that required high dose corticosteroid therapy and IVIG. Since the presence of distant metastases, first line therapy was started after palliative cystectomy. During chemotherapy DM symptoms initially improved, presumably due to IVIG and steroid therapy administrated during each cycle of chemotherapy. Subsequently, pembrolizumab was well tolerated, probably due to IVIG treatment that contributed to avoid DM aggravation, even though disease response to treatment might also have a role. After 6 months, DM symptoms rapidly worsened requiring higher dose of steroids; at the same time CT scan showed cancer progression. Therefore, paraneoplastic DM and underlying neoplasm showed a parallel clinical course through entirely medical history, as represented in **Figure 2**: after an initial improvement due to high-dose steroid and IVIG, clinical manifestations of DM rapidly worsened concomitantly with cancer diagnosis; at this point, we observed another amelioration immediately after chemotherapy administration, and DM symptoms decreased until reaching a plateau; DM manifestations remained mild also during the first 6 months of immunotherapy and restarted to get worse concomitantly with liver metastasis growth. Pembrolizumab was discontinued owing to disease progression and not to toxicity, and the absence of other irAEs and ocular findings (oculomotor involvement is typically associated with immunotherapy-related myositis) seem to corroborate this hypothesis, although a role in autoimmune paraneoplastic syndrome exacerbation cannot be totally ruled out. To the best of our knowledge, there are only few other cases reporting administration of ICIs in patients with preexisting paraneoplastic DM (22–24) (**Table 1**). Estenaga A et al. described a 78-year-old patient treated with chemoimmunotherapy (cisplatin + etoposide + atezolizumab) for advanced small cell lung cancer, while Poli De Frias F et al. reported a case of gastric adenocarcinoma in a 49-year-old male patient that received chemotherapy + nivolumab as first line regimen. Both patients showed an exacerbation of paraneoplastic DM during ICIs administration demanding high-dose steroids; atezolizumab was permanently discontinued in the first case, while the other patient continued nivolumab in association with chemotherapy and then alone as maintenance therapy (22, 23). Kim S et al. presented two cases of metastatic small cell lung cancer patients receiving the same first line therapy (carboplatin + etoposide + atezolizumab) albeit with different outcomes. In the first patient systemic treatment achieved not only tumor response but also a concomitant improvement of muscle strength and skin manifestation attributable to cancer-related DM; the other case, conversely, developed severe skin changes and deterioration of general condition until death, suggesting a fatal irAEs rather than an aggravation of paraneoplastic DM (24). Finally, our patient showed a severe exacerbation of skin and muscular manifestation after the first administration of EV. This first-in-class Nectin-4-directed antibody-drug conjugate has been associated to dermatologic adverse reactions (even fatal events) due to Nectin-4 expression in epidermal keratinocytes and skin appendages (25). However, the concomitant presence of muscular weakness, dysphagia and severe fatigue might indicate an aggravation of paraneoplastic DM, potentially due to disease progression either than a treatment toxicity.

**Table 1:** Reported cases of immune checkpoint inhibitors in patients with preexisting paraneoplastic dermatomyositis.

Reference [year]	Study type	Age and gender	Time of DM onset	Cancer type and staging	Cancer treatment (with ICIs)	DM during cancer treatment	Treatment for DM	ICIs discontinuation
Estenaga A. et al. (22)	CR	78 yo M	Months before the cancer diagnosis	Advanced small-cell lung cancer	Cisplatin + Etoposide + Atezolizumab	Worsening	High-dose steroids, topical steroids	Yes
Poli De Frias F. et al. (23)	CR	49 yo M	Concomitant to cancer diagnosis	Locally advanced gastric adenocarcinoma	FLOT + Nivolumab (added from 3^rd^ cycle)	Worsening	High-dose steroids	No
Kim S et al. (24)	CR	78 yo M (patient A)	Concomitant to cancer diagnosis	Advanced small-cell lung cancer	Carboplatin + Etoposide + Atezolizumab	Improving	None	No
Kim S et al. (24)	CR	57 yo M (patient B)	Concomitant to cancer diagnosis	Advanced small-cell lung cancer	Carboplatin + Etoposide + Atezolizumab	Worsening ()?	Antibiotics	Yes

CR, case report; M, male, F, female; FLOT, fluorouracil, leucovorin, oxaliplatin, and docetaxel.

## Conclusions

In conclusion, the role of ICIs administration in patients with preexisting cancer-related DM still appears challenging due to the higher risk of symptoms exacerbation and irAEs onset. In the absence of data coming from pivotal trials, real world experiences might help physicians in differential diagnosis between paraneoplastic syndrome and treatment-related adverse events occurring throughout systemic therapy with ICIs. Moreover, case reports focusing on this specific population might provide significant information regarding the management of immunotherapy and cancer-related DM. On the basis of our experience and other reported cases, previous paraneoplastic DM might not necessarily represent an absolute contraindication for ICIs treatment, with a strong correlation between antitumor treatment efficacy and DM improvement. Obviously, patients require a strict monitoring and a prompt administration of high-dose steroids and eventually other immunosuppressive agents in case of DM worsening. Lastly, our case might suggest a role of IVIG administration (previously and simultaneously to immunotherapy) in preventing DM reactivation although further evidence is needed to routinely recommend this procedure.

## Data Availability

The original contributions presented in the study are included in the article/**Supplementary Material**. Further inquiries can be directed to the corresponding author.
