# Supplementary material for: Case Report: Pembrolizumab in a patient with preexisting paraneoplastic dermatomyositis and sarcomatoid urothelial carcinoma. Searching for balance
Source: Front Immunol. 2025 Mar 17;16:1558964. doi: 10.3389/fimmu.2025.1558964 (PMC11955501; doi:10.3389/fimmu.2025.1558964)
Supplement: Supplementary file 1 [file DataSheet1.pdf]

CARE case report guidelines

CARE Checklist of information to include when writing a case report

(cc) BY-NC-ND

Topic	Item	Checklist item description	Reported on Line
Title	1	The diagnosis or intervention of primary focus followed by the words "case report"	1-2
Key Words	2	2 to 5 key words that identify diagnoses or interventions in this case report, including "case report"	23-24
Abstract (no references)	3a	Introduction: What is unique about this case and what does it add to the scientific literature?	29-32
	3b	Main symptoms and/or important clinical findings	32-34
	3c	The main diagnoses, therapeutic interventions, and outcomes	32-34
	3d	Conclusion—What is the main "take-away" lesson(s) from this case?	34-37
Introduction	4	One or two paragraphs summarizing why this case is unique (**may include** references)	40-54
Patient Information	5a	De-identified patient specific information	57
	5b	Primary concerns and symptoms of the patient	58-61
	5c	Medical, family, and psycho-social history including relevant genetic information	57
	5d	Relevant past interventions with outcomes	
Clinical Findings	6	Describe significant physical examination (PE) and important clinical findings	58-61
Timeline	7	Historical and current information from this episode of care organized as a timeline	figure 2
Diagnostic Assessment	8a	Diagnostic testing (such as PE, laboratory testing, imaging, surveys)	61-65
	8b	Diagnostic challenges (such as access to testing, financial, or cultural)	
	8c	Diagnosis (including other diagnoses considered)	61-65
	8d	Prognosis (such as staging in oncology) where applicable	
Therapeutic Intervention	9a	Types of therapeutic intervention (such as pharmacologic, surgical, preventive, self-care)	66-68; 78-108
	9b	Administration of therapeutic intervention (such as dosage, strength, duration)	66-68; 78-108
	9c	Changes in therapeutic intervention (with rationale)	78-108
Follow-up and Outcomes	10a	Clinician and patient-assessed outcomes (if available)	78-108
	10b	Important follow-up diagnostic and other test results	78-108
	10c	Intervention adherence and tolerability (How was this assessed?)	78-108
	10d	Adverse and unanticipated events	78-108
Discussion	11a	A scientific discussion of the strengths AND limitations associated with this case report	181-191
	11b	Discussion of the relevant medical literature **with references**	192-204
	11c	The scientific rationale for any conclusions (including assessment of possible causes)	212-223
	11d	The primary "take-away" lessons of this case report (without references) in a one paragraph conclusion	212-223
Patient Perspective	12	The patient should share their perspective in one to two paragraphs on the treatment(s) they received	84
Informed Consent	13	Did the patient give informed consent? Please provide if requested	Yes x No ☐
